# Lung tissue remodelling in MCT-induced pulmonary hypertension: a proposal for a novel scoring system and changes in extracellular matrix and fibrosis associated gene expression

**DOI:** 10.18632/oncotarget.13220

**Published:** 2016-11-08

**Authors:** Marcus Franz, Katja Grün, Stefan Betge, Ilonka Rohm, Bernadin Ndongson-Dongmo, Reinhard Bauer, P. Christian Schulze, Michael Lichtenauer, Iver Petersen, Dario Neri, Alexander Berndt, Christian Jung

**Affiliations:** ^1^ Department of Internal Medicine I, Jena University Hospital, Jena, Germany; ^2^ Department of Angiology, Cardiovascular Center Bad Bevensen, Bad Bevensen, Germany; ^3^ Institute of Molecular Cell Biology, Center for Molecular Biomedicine, Jena University Hospital, Jena, Germany; ^4^ Clinic of Internal Medicine II, Department of Cardiology, Paracelsus Medical University of Salzburg, Austria; ^5^ Institute of Pathology, Jena University Hospital, Jena, Germany; ^6^ Department of Chemistry and Applied Biosciences, Swiss Federal Institute of Technology (ETH Zürich), Zurich, Switzerland; ^7^ Department of Internal Medicine, Division of Cardiology, Pulmonology and Vascular Medicine, Heinrich-Heine-University, Düsseldorf, Germany

**Keywords:** pulmonary hypertension, histological score, remodeling, extracellular matrix, fibronectin

## Abstract

Pulmonary hypertension (PH) is associated with vasoconstriction and remodelling. We studied lung tissue remodelling in a rat model of PH with special focus on histology and extracellular matrix (ECM) remodelling. After induction of PH by monocrotaline, lung tissue was analysed histologically, by gene expression analysis and immunofluorescence labelling of ED-A domain containing fibronectin (ED-A^+^ Fn), B domain containing tenascin-C (B^+^ Tn-C) as well as alpha-smooth muscle actin (α-SMA). Serum concentrations of ED-A^+^ Fn were determined by ELISA. Systolic right ventricular pressure (RVPsys) values were significantly elevated in PH (*n* = 18; 75 ± 26.4 mmHg) compared to controls (*n* = 10; 29 ± 19.3 mmHg; *p* = 0.015). The histological sum-score was significantly increased in PH (8.0 ± 2.2) compared to controls (2.5 ± 1.6; *p* < 0.001). Gene expression analysis revealed relevant induction of several key genes of extracellular matrix remodelling. Increased protein deposition of ED-A^+^ Fn but not of B^+^ Tn-C and α-SMA in lung tissue was found in PH (2.88 ± 3.19 area%) compared to controls (1.32 ± 0.16 area%; *p* = 0.030). Serum levels of ED-A^+^ Fn were significantly higher in PH (*p* = 0.007) positively correlating with RVPsys (r = 0.618, p = 0.019). We here present a novel histological scoring system to assess lung tissue remodelling in PH. Gene expression analysis revealed induction of candidate genes involved in collagen matrix turnover, fibrosis and vascular remodelling. The stable increased tissue deposition of ED-A^+^ Fn in PH as well as its dynamics in serum suggests a role as a promising novel biomarker and potential therapeutic target.

## INTRODUCTION

Pulmonary hypertension (PH) is defined as an elevation of the mean pulmonary artery pressure (PAP) above 25 mmHg and can be divided into several clinical groups, which differ not only in pathogenesis but also patients' prognosis as well as treatment strategies [1, 2]. Common frequent groups are pulmonary arterial hypertension (PAH), PH due to left heart disease, PH due to lung disease and/or chronic hypoxia and chronic thromboembolic PH (CTEPH) [3]. The onset of PH is regularly associated with a markedly impaired prognosis and the therapeutic options are limited. Hitherto there is no curative treatment strategy being capable to stop or even reverse the disease. Specific therapies are only available for PAH (e.g., prostanoids, endothelin receptor antagonists, phosphodiesterase inhibitors). For all other groups of PH, except CTEPH in which a specific treatment (soluble gcp agonist) has been recently approved, therapy is limited to symptom control or treatment of the underlying disease, mostly cardiovascular or pulmonary disorders [4]. Despite of specific treatment options for PAH patients, mortality rates are still high and clinical management therefore needs to be further improved. Against that background, there is a certain scientific and especially clinical need to identify first) novel biomarkers for the early detection of PH and PAH as well as for valid prognosis estimation and second) novel targets for specific molecular therapies not only for PAH but also for the other PH groups.

The complex histological changes in lung tissue occurring in association to PH or PAH have only marginally been studied some decades ago. Additionally, the suggested scoring systems exclusively focus on vascular remodelling in patients suffering from congenital heart disease [5–7]. Thus, the findings are not conferrable to PH or PAH as common final paths evolving from different aetiologies. Anyhow, there is no established system to quantitatively assess the PH associated histological features in its entirety.

Besides vasoconstriction and thrombosis, the complex process of pulmonary vascular remodelling is a key component in the pathogenesis of PH and PAH [2]. Vascular remodelling involves both arterial and venous vessel structures and is characterised by an activation and proliferation of all cellular components, in particular endothelial cells of the tunica intima, vascular smooth muscle cells of the tunica media as well as adventitial fibroblasts. Mainly by an accumulation of activated fibro-/myofibroblasts in spatial association to the endothelial cell layer with a resulting synthesis and stromal deposition of extracellular matrix (ECM) components, the so-called neointima occurs. Within the tunica media, smooth muscle cells become activated and, together with myofibroblasts derived from the adventitia, transmigrate in luminal direction to the endothelial cell layer. As a result of this complex interplay between cells and ECM components, a concentric hyperplasia of the tunica intima develops which distinctly differs from classic atherosclerosis in which eccentric plaque formation is typical [8]. Over time, a progressive vessel occlusion occurs and represents the histo-pathologic substrate of PAP elevation leading to the clinical manifestations of the disease [2, 9]. The histo-pathological features of pulmonary vascular remodelling resemble the phenomenon of allograft vasculopathy (CAV) as it occurs in cardiac transplants [2, 8]. In CAV, reorganisation of the ECM, in particular the re-expression of fetal variants of certain cell adhesion modulating proteins like fibronectin or tenascin-C as well as the impact of fibroblast to myofibroblast transdifferentiation and vascular smooth muscle cell activation has been extensively described [10–14]. Thus, it can be assumed that these vascular ECM reorganisation processes also matter in pulmonary vascular remodelling in the PH development. Against that background, a deep analysis of ECM and fibrotic remodelling as well as MyoFb and VSMC activation is also promising in PH with respect to the identification of novel biomarkers for diagnosis and prognosis estimation as well as target molecules for innovative future strategies for specific therapeutic modulation of vascular remodelling in the lung. The value of fetal ECM components, mostly Tn-C variants, as biomarkers, both in tissue and in circulating blood, in a variety of cardiovascular diseases has been recently proven [15–20]. Due to its stable extracellular deposition, fetal variants of fibronectin or tenascin-C, in particular ED-A domain containing Fn (ED-A^+^ Fn) as well as A1 or B domain containing Tn-C (A1^+^ Tn-C, B^+^ Tn-C) are excellent molecular targets for antibody based delivery of diagnostic (e.g., radionuclides) or therapeutic agents (bioactive payloads, e.g. cytokines; or classical drugs) directly to the site of pathologic remodelling where the antigen is abundantly expressed while sparing normal tissue where the antigen is virtually absent. Such agents, in particular immunocytokines or antibody-drug conjugates, have been successfully administered in a variety of animal models of neoplastic and also non-neoplastic chronic-inflammatory disease [21, 22]. At least in part, such immunocytokines have already reached the stage of clinical testing [23–26].

In contrast to several fetal Tn-C variants, for ED-A^+^ Fn there are only very limited data concerning its potential impact as serum biomarker in cardiovascular diseases and, to our best knowledge, there is currently no study available in the literature reporting on the role of that molecule as potential biomarker in pulmonary hypertension.

### Aims of the study

Motivated by the considerations described above and based on recent studies of our group, the current study was aimed to analyse lung tissue alterations in an appropriate animal model of induced pulmonary hypertension (IPH). Therefore, especially histological, extracellular matrix and fibrosis-associated remodelling in IPH rats has been studied in detail. Furthermore, the expression of certain fetal variants of Fn (ED-A^+^ Fn) and Tn-C (B^+^ Tn-C) and also alpha-smooth muscle actin (α-SMA) as a marker for activated fibroblasts/myofibroblasts and vascular smooth muscle cells have been investigated. To test ED-A^+^ Fn as a novel biomarker of the disease, its serum concentrations has been determined by ELISA.

## RESULTS

### Characterization and haemodynamic validation of the animal model of IPH

**Figure 1:**
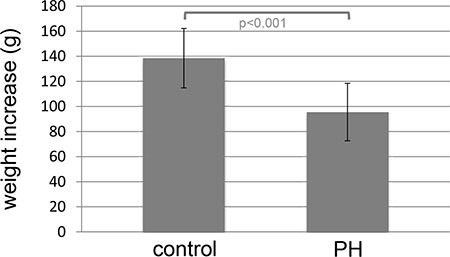
Higher increase in body weight in healthy controls (control) rats (*n* = 10) compared to MCT induced pulmonary hypertension (IPH) rats (*n* = 18)

All 28 rats showed a continuous increase in body weight but the level was higher in healthy control (*n* = 10) compared to IPH (*n* = 18) rats (*p* < 0.001; Figure 1).

Moreover clinical conditions of the animals were assessed daily. In general, one could observe that IPH rats developed clinically manifest dyspnoea, which increased over time. At day 8 after MCT injection, the IPH rats showed increased parameters of the above-described clinical severity score compared to the control rats.

**Figure 2:**
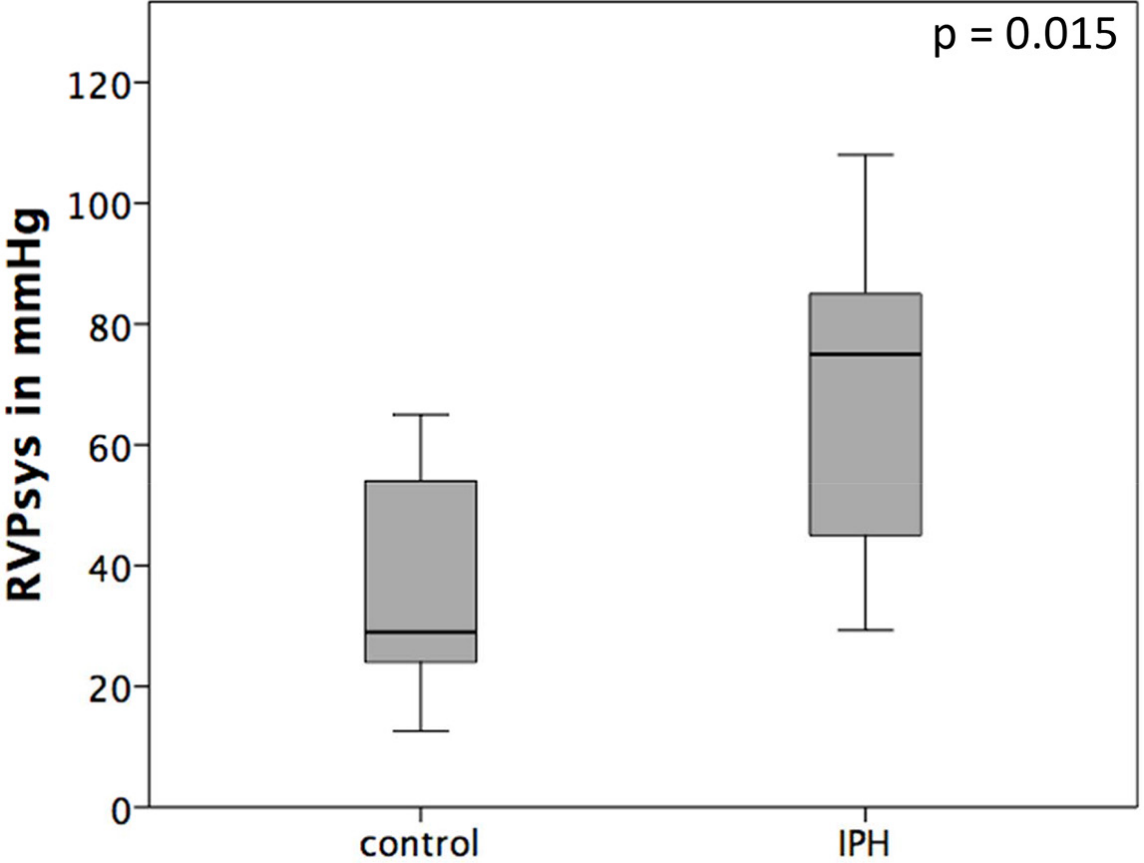
Haemodynamic results after right heart catheterization The RVPsys is significantly elevated in MCT induced pulmonary hypertension (IPH) rats compared to control rats (control).

In all IPH rats, the RVPsys (RVPsys = 75 ± 26.4 mmHg) could be shown to be significantly elevated compared to control rats (29 ± 19.3 mmHg) (*p* = 0.015; Figure 2).

### Histological analysis of lung tissue and development of a novel scorings system to assessment of pulmonary tissue damage in IPH

**Figure 3:**
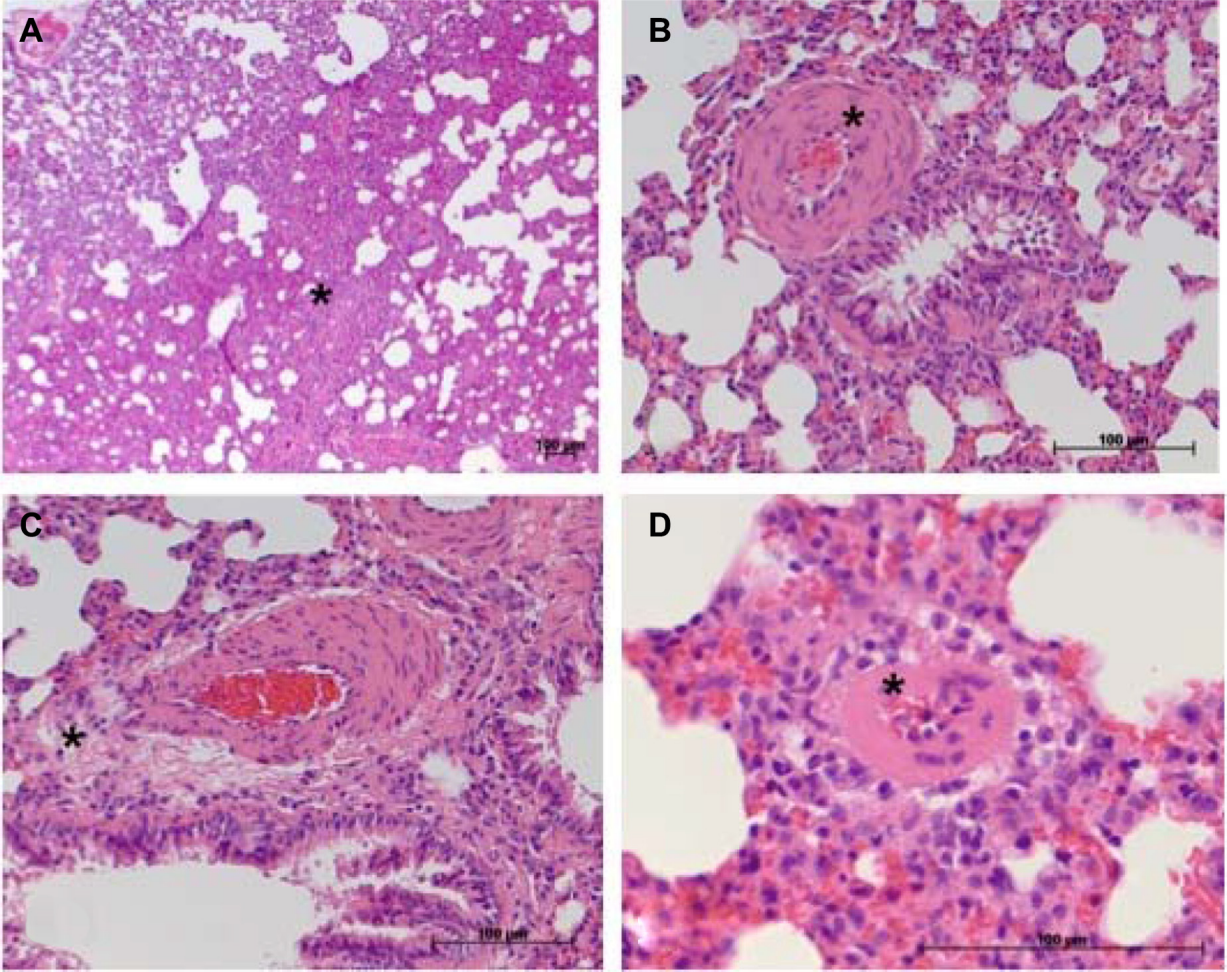
Considerable histopathological features occurring in damaged lung tissue in the rat model of IPH contributing to the sum-score system (**A**) atelectasis area (*, here ≥ 30% of total tissue section area); (**B**) moderate media hypertrophy of a peribronchial artery (*); (**C**) perivascular cellular edema surrounding a peribronchial artery; (**D**) at least moderate media hypertrophy of a small artery (*) not showing spatial association to a bronchus structure.

**Figure 4:**
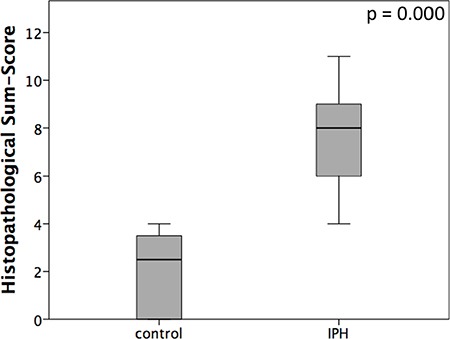
Graphical presentation of histopathological sum-scores assessed using the described scoring system in healthy controls (control) rats compared to MCT induced pulmonary hypertension IPH rats

Representative samples of the histopathological scoring parameters included in the sum-score system to quantify the extent of lung tissue damage in IPH rats are given in Figure 3. Finally, for each animal (10 controls and 18 IPH rats) a sum-score value could be calculated. Compared to the control rats showing normal lung tissue (sum-score 2.5 ± 1.6), all 18 IPH rats exhibited distinct signs of lung damage - the sum-score values were significantly elevated (8.0 ± 2.2; *p* < 0.001) as shown in Figure 4.

### RT-PCR based gene expression profiling of fibrosis as well as extracellular matrix and adhesion molecules

**Figure 5:**
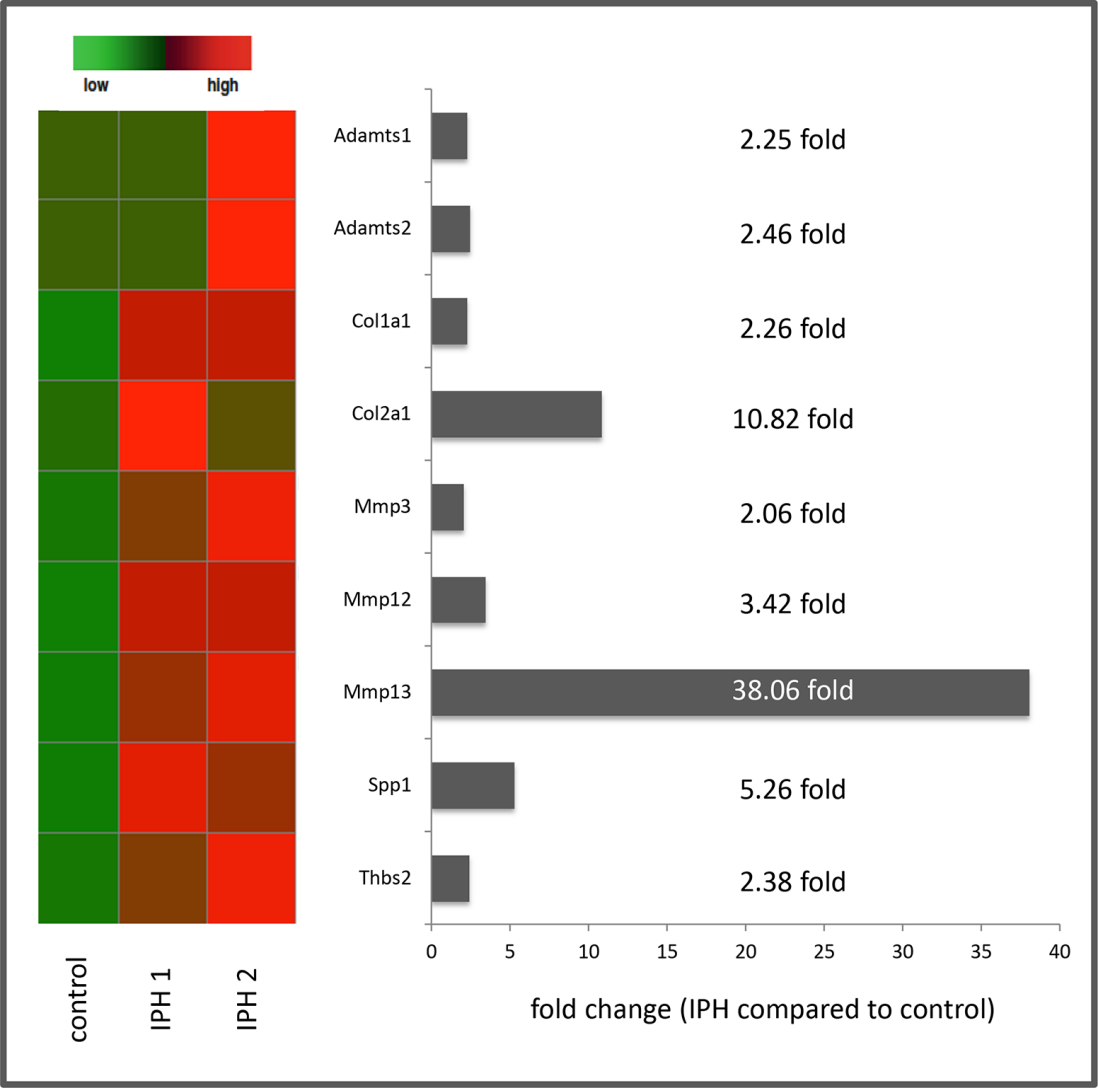
Summary of the results of PCR based gene expression analysis with heat map presentation of relevantly (> 2 fold) up-regulated genes as well as fold changes of gene expression in MCT induced pulmonary hypertension (IPH) compared to control rats

Pathway-focused PCR-based gene expression analysis of ECM and Adhesion Molecules as well as Fibrosis-associated gene arrays (84 genes each) revealed relevant (> 2 fold) up-regulations for the following 9 genes: a disintegrin and metalloproteinase with a thrombospondin type 1 motif, member 1 (ADAMTS 1) (2.25 fold), a disintegrin and metalloproteinase with a thrombospondin type 1 motif, member 2 (ADAMTS 2) (2.46 fold), collagen type I alpha 1 (2.26 fold), collagen type II alpha 1 (10.82 fold), matrix metallopeptidase 3 (MMP 3) (2.06 fold), matrix metallopeptidase 12 (MMP 12) (3.42 fold), matrix metallopeptidase 13 (MMP-13) (38.06 fold), Osteopontin (gen: secreted phosphoprotein 1 = spp1) (5.26 fold) and Thrombospondin 2 (Thbs2) (2.38 fold) (Figure 5).

Interestingly, despite of the high number of genes included in the pathway-focused arrays performed in this study, we could not observe any relevant (> 2 fold) down-regulations of ECM or fibrosis associated genes in the rat model of induced IPH.

### Protein deposition and tissue distribution of ED-A^+^ Fn, B^+^ Tn-C and α-SMA

Among the fetal splicing variants of Fn and Tn-C, in particular ED-A^+^ Fn and B^+^ Tn-C have been shown to play a pivotal role in vascular and interstitial tissue remodelling in a variety of diseases. α-SMA is a marker for activated vascular smooth muscle cells (VSMCs) and myofibroblasts (MyoFb) both representing crucial cell phenotypes driving vasculopathy and fibrosis [14]. Thus, these three markers have been studied in detail in the rat model of IPH using immunofluorescence labelling and cLSM based qualitative and semi-quantitative tissue distribution analysis.

**Figure 6:**
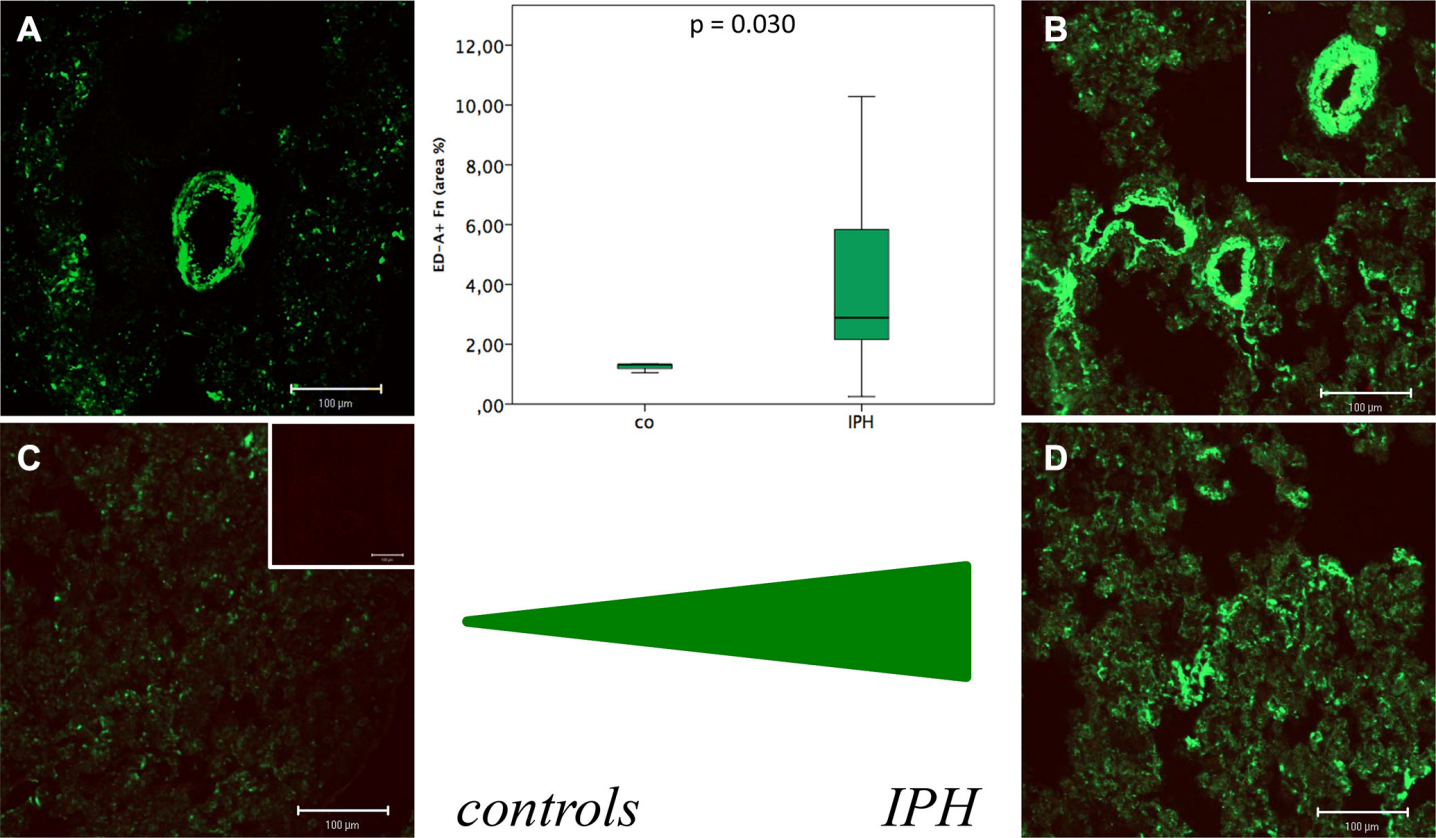
Immunofluorescence labelling of ED-A^+^ Fn (green fluorescence) in lung tissue of control (A and C) and MCT induced pulmonary hypertension (IPH) (B and D) rats in spatial association to vessel structures (A and B) and, to a lesser extent, lung parenchyma (C and D) as well as results of quantitative protein deposition analysis (boxplot presentation)

Immunofluorescence labelling of ED-A^+^ Fn (green fluorescence) revealed a relevant expression and extracellular deposition in lung tissue of both, control (Figure 6A and 6C) and IPH (Figure 6B and 6D) rats in spatial association to vessel structures (Figure 6A and 6B) and, to a lesser extent, lung parenchyma (Figure 6C and 6D) as well. Quantitative analysis showed a significant increase in the total amount ED-A^+^ Fn protein deposition in lung tissue of PH (2.88 ± 3.19 area%) compared to control rats (1.32 ± 0.16 area%; *p* = 0.030, Figure 6).

**Figure 7:**
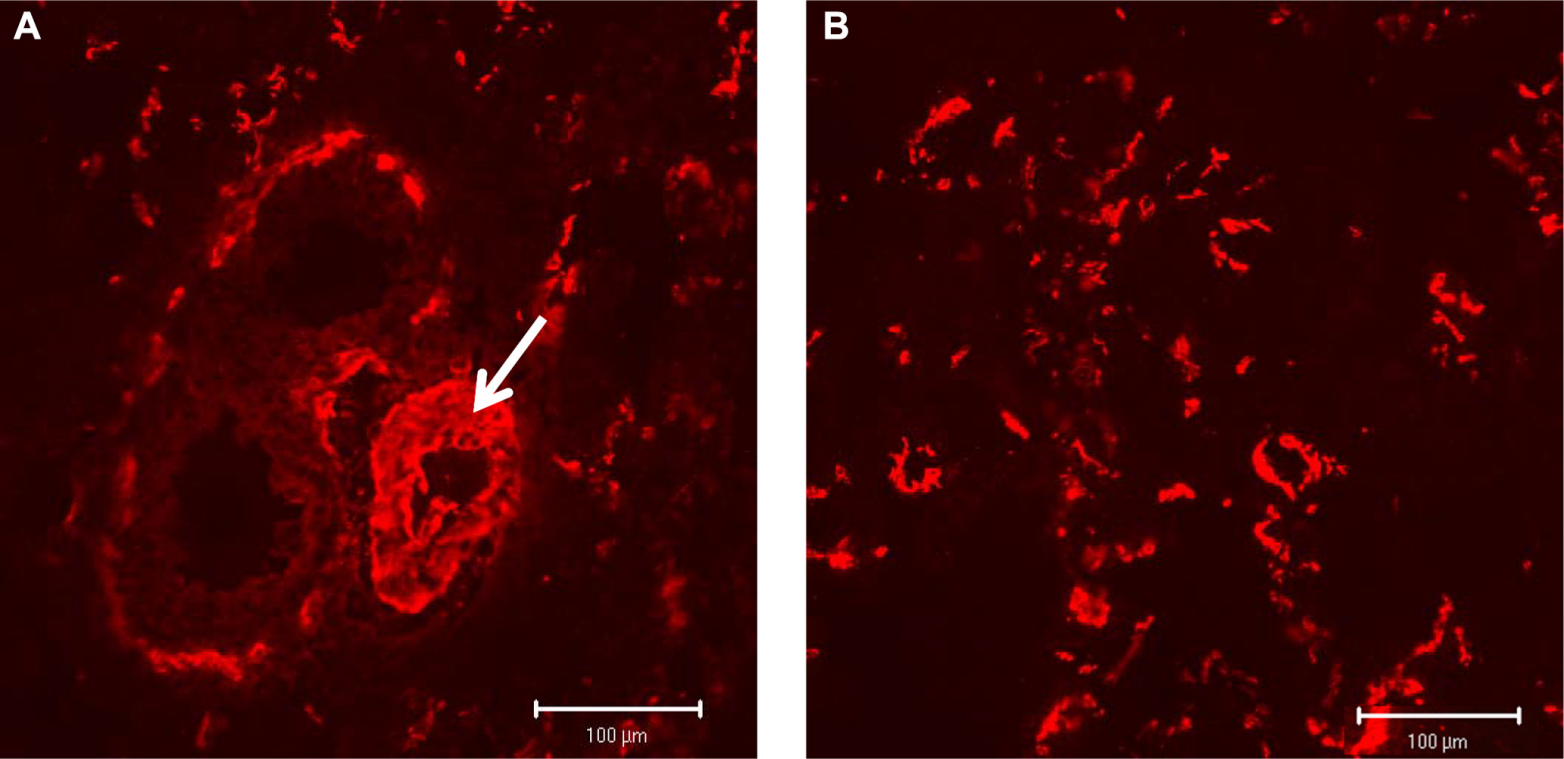
Immunostaining of α-SMA (red fluorescence) showing a protein expression in association to larger vessel structures (A, white arrow) as well as in lung stroma (B)

In contrast, for B^+^ Tn-C only very low tissue expression levels could be shown in con rats without any changes in IPH rats. Because of the low expression, a cLSM based quantitative analysis has not been performed for B^+^ Tn-C. Immunostaining of α-SMA (red fluorescence) demonstrated a protein expression in association to larger vessel structures containing a tunica media, in which VSMCs are positive for the protein (Figure 7A, white arrow), as well as in lung stroma where activated fibroblasts/MyoFb exhibit α-SMA positivity (Figure 7B). Quantitative analysis did not reveal significant differences in the total amount of α-SMA protein expression between PH (2.59 ± 1.32 area%) and control rats (2.79 ± 0.224 area%, *p* = 0.464).

### Quantification of ED-A^+^ Fn serum levels

**Figure 8:**
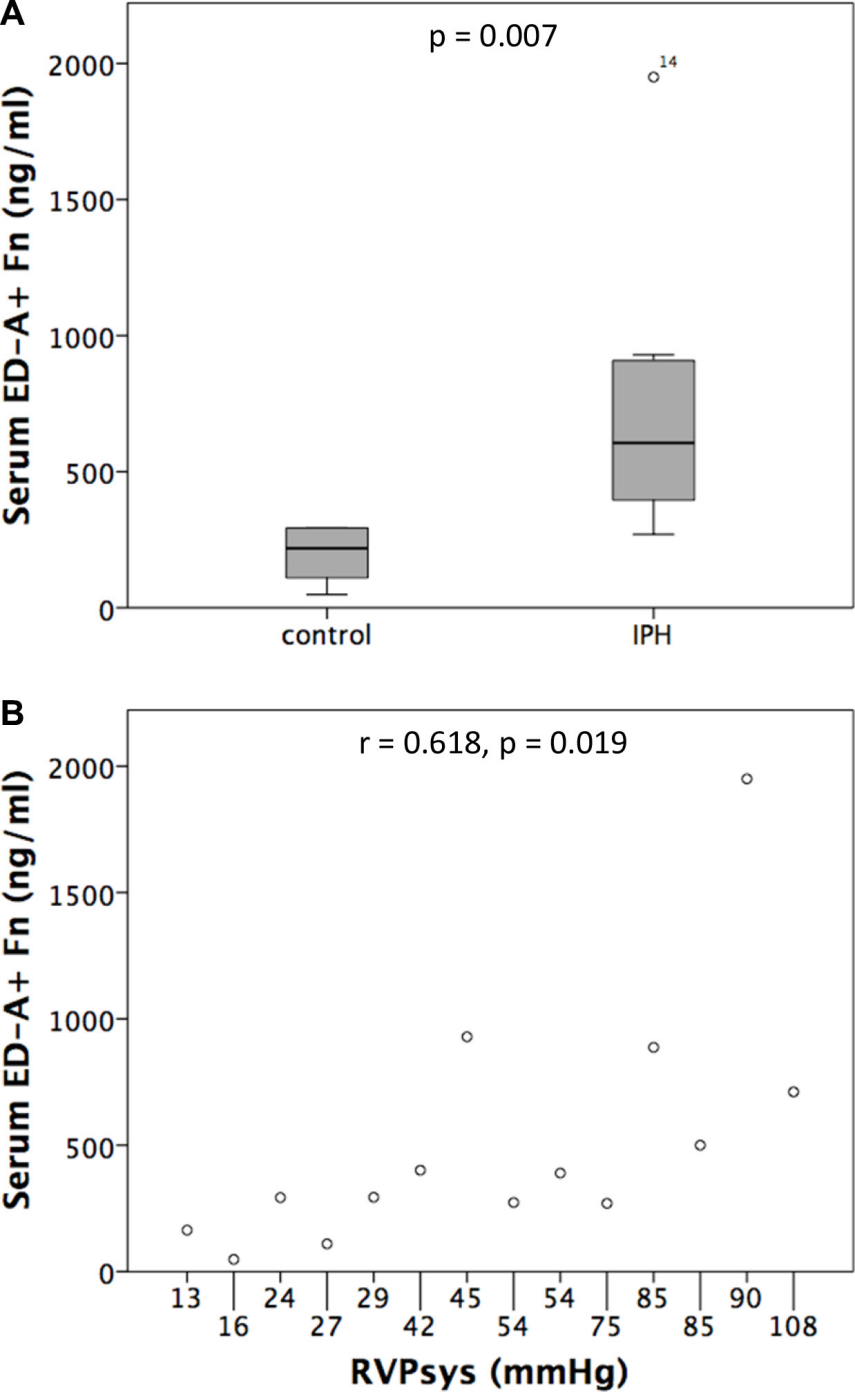
Serum levels of ED-A^+^ Fn determined by ELISA showed a significant increase in MCT induced pulmonary hypertension (IPH) compared to control rats (A) and a positive correlation to RVPsys values (B)

Considering the increased protein expression of ED-A^+^ Fn in PH compared to control rats and the fact that a liberation of ED-A^+^ Fn into the circulation in human diseases has been described before, we performed serum quantification of ED-A^+^ Fn by ELISA and compared control (*n* = 6) with IPH rats (*n* = 8). A specific and quantitative relevant serum concentration of ED-A^+^ Fn could be detected in all rats investigated. The serum levels of the protein were markedly increased in IPH (605.5 ± 539.46 ng/ml) compared to control (218.5 ± 105.15 ng/ ml) rats (*p* = 0.007, Figure 8A). Moreover, correlation analysis revealed a positive correlation between RVPsys values and serum ED-A^+^ Fn concentrations (r = 0.618, *p* = 0.019, Figure 8B).

## DISCUSSION

The present study was focused on the investigation of lung tissue remodelling in PH, in particular with respect to the analysis of the complex histo-pathological changes as well as ECM and fibrosis-associated remodelling processes. The applied rat model of MCT induced PH, which is an established approach [27], has been validated by haemodynamic analysis.

Since PH is a process not only involving pulmonary vessel structures but also the entire lung tissue, a novel sum-scoring system to assess PH associated alterations also in lung parenchyma and stroma as well is, suggested by us. The score includes all relevant histological features available from the literature [5] and obviously occurring during IPH development in adequate animal models. We could demonstrate that IPH rats reproducibly exhibit significantly increased score values compared to controls. Until now, such a scoring system does not exist - may be due to the restricted availability of human lung tissue. The only scoring systems that can be found in the literature have been developed in patients suffering from congenital heart disease some decades ago and exclusively focus on the characterization of vascular remodelling processes [6, 7]. The novel score suggested here, could build the basis for integrative functional research approaches including not only pulmonary vessels but also lung parenchyma and stroma and is therefore filling a scientific gap. But of course, and this is a limitation that should be mentioned here, the sum-score has to be validated externally using a different animal model, e.g., a model of hypoxia induced PH. This should be the object of further studies.

By mRNA expression analysis, several interesting candidate genes has been identified possibly being functionally involved in the process of PH associated tissue remodelling. The 9 genes identified to be relevantly up-regulated during IPH development in our rat model are known to be crucially involved in - especially vascular - remodelling processes in IPH. Thus, Adamts1 plays a crucial role in various inflammatory processes and Adamts2 contributes to the processing of pro-collagen proteins [28]. The increased expression of coll1a1 and col2a1 in IPH compared to control rats observed in the current study goes in line with recent studies reporting on the importance of collagen metabolism in the development of PH also in the human system [29, 30]. We detected an up-regulation of Mmp3, Mmp12 and Mmp13 in IPH rats. The crucial role of the MMP/TIMP – system in PH has already been investigated in the past and a certain role of the proteolytic degradation of pre-existing ECM structures mediated by these enzymes has been highlighted. In these studies, in particular Mmp2, Mmp3 and Mmp9 were observed to be of functional relevance in MCT induced IPH [31]. An up regulation of the isoforms Mmp12 and Mmp13 as detected by us, has not been reported before and should be investigated in more detail in the near future. The increased expression of Osteopontin has been described in PH both as a potential biomarker in the human system [32] and a crucial functional mediator of the activation of adventitial fibroblasts in an animal model of hypoxia induced PH [33, 34]. For Thrombospondin2 there is only limited data, but the report about an up regulation in idiopathic pulmonary fibrosis associated PH compared to COPD associated PH [35] partially goes in line with our finding of an increased expression in MCT induced PH. Taken together, the 9 genes that could be evidenced to be relevantly regulated in our IPH rat model should be further evaluated as novel biomarkers or therapeutic targets to address pulmonary vascular but also whole organ remodelling in the process of PH development.

Since fetal vatriants of Fn and Tn-C could be recently shown to be stably deposited in the ECM during tissue remodelling and therefore qualify as excellent molecular targets for antibody based treatment strategies [23, 26] we investigated the expression of ED-A^+^ Fn and B^+^ Tn-C. In contrast to B^+^ Tn-C, for ED-A^+^ Fn a relevantly increased expression and stable deposition in spatial association to vessel structures and the lung parenchymal and stromal compartment was demonstrated in this study. Thus, the molecule can be suggested as a promising novel target molecule for antibody based imaging or pharmaco-delivery using the human recombinant antibody F8, which is specific for ED-A^+^ Fn. A wide range of immunocytokines containing this antibody and pro- or antiinflammatody cytokines is available. This might pave the way for completely novel approaches in the treatment of PH. To elucidate this, further pre-clinical studies using the MCT induced PH rat model should be performed in the near future. The principle of an F8 based targeted delivery of cytokines or contrast agents has been proven for cardiovascular disease by our group using a heterotopic rat heart transplantation model [21, 22]. Against the background of these findings and data from the literature [10], a central role for ED-A^+^ Fn and also fetal Tn-C variants has been postulated for the chronic cardiac rejection process with vasculopathy and fibrosis [14]. Since B^+^ Tn-C is only marginally expressed in the rat model of induced PH, shown in the current study, one could assume that this Tn-C variant might not be of certain importance in the process of PH development. However, to draw such a far-reaching conclusion, further extended studies must be carried out. Moreover, in the current study we could not show differences in the protein expression level of α-SMA between PH and control rats. This result is rather surprising since one could have hypothesized that at least the pulmonary vascular remodelling process is accompanied by an activation and proliferation of VSMCs as it has been shown for other cardiovascular disorders like cardiac allograft vasculopathy [8].

By reason of the extensive re-expression of ED-A^+^ Fn in lung tissue of IPH compared to control rats, the question arises, if the molecule, especially due to its distinct vascular deposition and accessibility from the blood stream, is liberated into the circulation and thereby might potentially serve as a valuable serum biomarker of the disease. To elucidate this, an ELISA protocol established by our group and capable of detecting the human as well as murine ED-A^+^ Fn, has been performed to measure serum concentrations in IPH compared to control rats. There were significantly higher ED-A^+^ Fn serum levels in diseased rats with a correlation to RVPsys. Thus, ED-A^+^ Fn originating from altered lung vessels or, as an alternative hypothesis, from the right ventricle which faces pressure overload and undergoes pathologic remodelling in PH, seems to be liberated into the circulation and might qualify as a promising biomarker not only for diagnosis of the disease but especially for patients' surveillance and monitoring of therapy response. To elucidate this, the marker should be evaluated in a large collective of patients suffering from PH of different aetiologies, especially in comparison between group 1 (PAH) and the other groups of PH [4]. Although a variety of novel biomarkers for PAH/PH have been identified and tested in the recent past [32], a reliable and specific serum biomarker especially for evaluation of therapy response and frequent risk-re-stratification is not available until now. ED-A^+^ Fn as a serum biomarker of vascular diseases including cardiovascular disorders and especially PH has not been investigated until now. Thus, the current study is the first one addressing this question for PH using an appropriate animal model. But, for other diseases, i.e., different malignancies and also non-neoplastic chronic-inflammatory disorders [36–44], the molecule had been suggested as a potential biomarker reflecting the state of disease in the broadest sense.

### Summary/highlights of the study

In summary, the current study provides novel data on lung tissue remodeling in an appropriate animal model of PH. A comprehensive histological sum-score system assessing not only pulmonary vascular but also parenchymal or stromal remodeling in the lung has been developed and tested in the animal model. This scoring system can be recommended as a worthwhile endorsement of lung tissue analysis in basic science as well as preclinical treatment studies using the animal model of MCT induced PH or similar approaches. But, before doing so, the scoring system has to be validated externally using a different animal model, e.g., a model of hypoxia induced PH. By identifying interesting novel candidate genes, the current study gives reason for further studies elucidating their functional role in the process of PH development as well as testing their value as biomarker or even therapeutic target of the disease. The fetal Fn variant ED-A^+^ Fn could be identified as a promising target for antibody based delivery of diagnostics as well as bioactive payloads (immunocytokines) or antibody-drug-conjugates (ADCs) directly to the site of disease. Here, especially the targeted delivery of anti-inflammatory and anti-proliferative cytokines, e.g., interleukin-10, seems to be of potential interest for further treatment studies. Moreover, a liberation of the molecule into the circulation has been proven and the serum levels measured by ELISA seem to reflect the extent of PH and associated tissue remodeling in the lung.

## MATERIALS AND METHODS

### Animal model of induced pulmonary hypertension (IPH)

For the animal model of IPH, in total 28 Sprague-Dawley-rats (200–250 g), obtained from Charles River (Sulzfeld, Germany) were used. The animals were allowed acclimatizing for at least 7 days before experimental procedures started. During this time period, the rats had ad libitum access to food and water and were exposed to controlled light/dark cycles. To induce pulmonary hypertension, rats (*n* = 18) were injected with a single dose of monocrotalin (MCT; 60mg/kg body weight; 300 μl) (Carl Roth, Germany) subcutaneously. The control rats (*n* = 10) were injected with NaCl (300 μl). To prevent secondary infection as well as inflammatory lung-alteration, rats received Enrofloxacin (Baytril, WDT, Germany) 2.5% ad water from day 2 to 15 after injection (post injectionem, p.i.) of MCT. For clinical monitoring, rats were weight and examined twice weekly. The health state (clinical conditions) was assessed using a clinical severity score (CSS) from 1 to 5 (1 = no signs of illness/2 = low-grade/3 = mild-grade/4 = high grade/5 = dead). Criteria of the Scoring were estimation of spontaneous activity, reaction to exogenous stimuli and posture.

On day 21 p.i., rats were anaesthetized and right heart catheterization was performed as described below. Finally, rats were sacrificed in deep anaesthesia and analgesia and the organs were excised and immediately shock frozen in liquid nitrogen (stored at –80°C) or formalin-fixed and paraffin embedded. Moreover, for serum measurements, blood samples were taken and immediately centrifuged at 2383 g for 25 min. Supernatants were shock frozen and stored at −80°C until further analysis.

The study protocol was approved by the appropriate State Office of Food Safety and Consumer Protection (TLLV, Bad Langensalza, Thuringia; local registration number: 02–004/14). All experiments were performed in accordance with The Principles of Laboratory Animal Care (NIH publication VI. 25, No. 28, revised 1996), the current version of the German Law on the Protection of Animals and the guidelines for animals care.

### Haemodynamic measurements

Right heart catheterization (RHC) was performed via the right vena jugularis interna using an 1.4F micro conductance pressure-volume catheter (model SPR-839; Millar Instruments Inc) for continuous registration of right ventricular blood pressure and, if technically feasible, also pulmonary artery blood pressure traces in closed chest animals. Registration and analysis of pressure curves were performed using a PowerLab system (ADInstruments Ltd., Oxford, UK) connected to the Millar catheter.

### Histological evaluation/assessment of pulmonary tissue damage

Formalin fixed and paraffin embedded 4 μm lung tissue sections were subjected to conventional H&E-staining (HE). histological staining. Since there is no standardized accepted histological scoring system to asses whole lung tissue sections with respect to the grade of histological damage in the setting of PH in an animal model, we established a novel soring system including all important histological parameters occurring in human pulmonary hypertension in combination with the most striking tissue alterations observable in the currently established rat model. Thus, the single histological features included in the scoring system were assessed semi-quantitatively and a sum-score was calculated as given in Table 1. Tissue sections of the explanted lungs were analysed independently by two experienced scientists (a cardiologist and a pathologist).

**Table 1:** Semi-quantitative scoring-system to assess the severity of lung tissue damage in rats with monocrotaline-induced pulmonary hypertension (PH)

Histopathological features (parameter)	Description	Semiquantitative assessment (Score Value)
**atelectasis area**	% area of atelectasis related to total area of tissue section	not detectable = 0 points < 30% = 1 point ≥ 30% = 2 points
**emphysema area**	% area of emphysema related to total area of tissue section	not detectable = 0 points < 30% = 1 point ≥ 30% = 2 points
**media hypertrophy of peribronchial arteries**	cellular hypertrophy of the tunica media of arteries spatially associated to bronchial structures	not detectable = 0 points weak = 1 point moderate = 2 points severe = 3 points
**perivascular cellular edema of peribronchial arteries**	cellular edema located in the perivascular region around peribronchial arteries	not detectable = 0 points detectable = 2 points
**media hypertrophy of small arteries**	cellular hypertrophy of the tunica media of small arteries showing no spatial association to bronchial structures	not detectable = 0 points weak = 1 point moderate = 2 points severe = 3 points
**Sum-Score**		**Maximum = 12 points**

### RT-PCR based gene expression profiling

After maceration of rat lung tissue in liquid nitrogen, RNA extraction was performed using the Maxwell^®^16 LEV simplyRNA Tissue Kit and the Maxwell^®^16 Instrument (Promega Corporation, Madison, USA) according to the recommendations of manufacturer. After completing, the concentration of total RNA was measured using a spectrophotometer (NanoDrop, ND-1000, Peqlab, Germany). For cDNA synthesis, total RNA was pooled (one control group with 3 animals and two PH groups with 6 rats each) to obtain 500 ng total RNA. The synthesis reaction was performed with the RT^2^ First Strand Kit (SABiosciences, Qiagen GmbH, Hilden, Germany) according to the manufacturers instructions. Thereafter, cDNA concentrations of each sample were measured using a spectrophotometer (NanoDrop, ND-1000, Peqlab, Germany) and then stored at –20°C until further use. For gene expression profiling in lung tissue, the Rat Fibrosis and the Rat Extracellular Matrix & Adhesion Molecules RT^2^ Profiler PCR Array system (SABiosciences, Qiagen GmbH, Hilden, Germany; 84 genes each) was used according to the instructions of the manufacturer. The Bio-Rad MyiQ real-time PCR system (Bio-Rad Laboratories GmbH, München, Germany) was used to perform real-time PCR reaction with a two-step cycling program with 1 cycle of 95°C for 10 min to activate the HotStar DNA-polymerase, followed by 40 cycles of 95°C for 15 s and 60°C for 1 min to detect and record the SYBR-Green fluorescence from every well during the annealing step of each cycle. Melting curves were generated for quality control. First, the threshold cycle (Ct) for each well was determined using the instruments software (Bio-Rad Laboratories GmbH, München, Germany). Second, the Ct values of the control wells (Genomic DNA Control, Reverse Transcription Control and Positive PCR Control) were analysed. Third, a ΔCt value for each gene in each plate was calculated using the mean value of the five house keeping genes for normalization. For better data visualization, a heat map was generated using *matrix2png* online interface (available under http://www.chibi.ubc.ca/matrix2png/bin/matrix2png.cgi). The ΔΔCt method was applied to investigate regulations in mRNA expression in comparison of different samples. The pool of control rats (*n* = 3) was defined as normalization group. The two pools of PH rats (*n* = 6 each) were defined as experimental group. The ΔΔCt value was generated as follows: ΔΔCt = ΔCt (experimental group) - ΔCt (controls). In a next step, fold changes were calculated using the 2-ΔΔCt method (if > 1, the result was reported as fold up-regulation; If < 1, the negative inverse of the result was reported as fold down-regulation).

### Immunofluorescence labeling, image analysis and quantification of tissue protein deposition

For immunofluorescence labeling, 4 μm cryostat sections were fixed in ice-cold acetone for 10 min. For the detection of ED-A^+^ Fn and B^+^ Tn-C, the mouse monoclonal antibodies IST-9 (Santa Cruz Biotechnology, Inc., Heidelberg, Germany), and 4C8MS (IBL Hamburg, Germany) were biotinylated using the Animal Research Kit (ARK, Dako, Deutschland GmbH, Hamburg, Germany). After inhibition of non-specific staining due to endogenous biotin by applying the DAKO Biotin Blocking System (Dako Deutschland GmbH, Hamburg, Germany), the antibodies IST-9 (dilution 1:20) and 4C8MS (dilution 1:20) were applied to tissue and incubated for 60 minutes at room temperature. Antibody reactivity was then visualized using the tyramide based signal amplification system TSA™Kit#22 (HRP-Streptavidin and Alexa Fluor_488 tyramide) (Invitrogen GmbH, Karlsruhe, Germany). For the detection of α-SMA, the 1A4 antibody (Dako Deutschland GmbH, Hamburg, Germany, dilution: 1:20) was applied as primary antibody for 60 minutes at room temperature. After thorough rinsing, a Cy3 (red fluorescence) conjugated donkey-anti-mouse antibody (Jackson ImmunoResearch, USA) was used as secondary antibody and allowed to incubate for 45 minutes at room temperature. Antibody specificity control staining was performed in accordance but by replacing the primary antibodies (data not shown). In a last step, sections were mounted using Vectashield H1200 containing DAPI-stain (Linaris biologische Produkte GmbH, Wertheim-Bettingen, Deutschland) and stored at –20°C. Analysis of immunofluorescence labeling was performed by confocal laser scanning microscopy (cLSM) using an LSM 510 microscope (Zeiss, Jena, Germany). To analyze the amount of ED-A^+^ Fn and α-SMA in lung tissue sections, the quantification tool of the LSM 510 Software Rel. 3.2 (Zeiss) was used. Within each section, six non-overlapping areas were chosen and a photograph of each was taken applying standardized microscope settings: objective lens type: ZEISS, Plan-APOCHROMAT 20x/0.75 objective; optical thickness: 1.2 μm; bit depth of acquired images: 8-bit; measurement area: 460.70 μm x 460.70 μm. Using software based analysis, the number of red (Cy3) or green (Alexa-488) pixels per image was determined and the stained area was calculated in percent of the total area. By averaging these data, a mean value representing the amount of protein deposition per lung tissue sample was achieved. To eliminate non-specific background fluorescence, two randomly chosen areas of the negative control staining from each sample were quantified in accordance and the results (mean value) were subtracted from the mean value of stained area in each sample.

### Quantitative analysis of ED-A^+^ Fn serum levels by ELISA technique

Quantitative analysis of ED-A^+^ Fn in serum of IPH as well as control rats was performed by ELISA technique using gelatin for capturing of Fn and the ED-A^+^ Fn specific antibody IST-9 for detection of ED-A^+^ Fn [45]. The ELISA plates (96 well, high binding, F-Bottom ELISA Microplate, Greiner Bio-one GmbH, Frickenhausen, Germany) were coated with 300 μl 0.5% gelatin in PBS / well overnight at 4°C. The following steps were performed at 20°C, all samples were diluted in PBS. 50 μl of the protein / serum samples (diluted in PBS, range 1:2 to 1:8) were added and allowed to incubate for one hour. For the detection of bound ED-A^+^ Fn, the monoclonal antibody IST-9 (dilution: 1:200) Santa Cruz Biotechnology) was added and allowed to incubate for one hour. Thereafter, a highly absorbed biotinylated donkey-α-mouse antiserum (715–066–151, diluted 1:10000, Dianova GmbH, Hamburg, Germany) was added for one hour. In a next step, streptavidin-horseradishperoxidase (SA-HRPO, dilution: 1:330) was applied and allowed to incubate for 40 minutes. Detection was realized using H_2_O_2_ and o-phenylendiaminodihydrochloride (OPD, 2mg, Dako, Hamburg, Germany) as chromogenic substrate. 100 μl 0.5 M H_2_SO_4_ were used to stop the reaction and absorbance was determined at a wavelength of 490 nm using a Tecan Sunrise Reader (Tecan Group, Männedorf, Switzerland). The IST-9 antibody reagents were obtained from Sirius Biotech S.r.L. (Genoa, Italy, www.sirius-biotech.com).

### Statistical analysis

Data are expressed as median ± standard deviation (SD). Statistical analyses were performed with IBM SPSS statistics, version 22.0 (IBM Inc.). Kruskal-Wallis Test was used to test for significant differences of the analysed parameters between different experimental groups. Bi-variate correlations between parametric variables were tested by Pearson's correlation coefficient. A *p*-value ≤ 0.05 was defined to be statistically significant.
